# Upgrading of Lignocellulosic Biomass toward the Development of Advanced Carbon Materials for Environmental Applications

**DOI:** 10.1021/acsestengg.6c00212

**Published:** 2026-07-03

**Authors:** Miriam Navlani-García, David Salinas-Torres, Ting Zhang, Zhenfeng Bian, Hexing Li, Diego Cazorla-Amorós, Hiromi Yamashita

**Affiliations:** † Department of Inorganic Chemistry and Materials Institute, 16718 University of Alicante, Ap. 99, Alicante E-03080, Spain; ‡ MOE Key Laboratory of Resource Chemistry and Shanghai Key Laboratory of Rare Earth Functional Materials, College of Chemistry and Materials Science, 12544 Shanghai Normal University, No. 100, Guilin Road, Shanghai 200234, China; § Division of Materials and Manufacturing Science, Graduate School of Engineering, The University of Osaka, 2-1 Yamada-Oka, Suita, Osaka 565-0871, Japan

**Keywords:** biomass upgrading, biomass valorization, environmental remediation, energy applications, catalytic applications

## Abstract

The use of biomass in the energy landscape has aroused great interest because of the diversity of high-value-added products that can be obtained. Among them, carbon materials stand out for properties such as their high chemical and thermal stability, large specific surface area, and possibility of modifying their surface properties by introducing heteroatoms, which make them ideal candidates for countless applications. The present review addresses to some of the representative works related to the preparation of advanced carbon materials prepared from biomass residues for applications of environmental interest, such as environmental remediation, catalytic processes, and energy-related devices.

## Graphical abstract

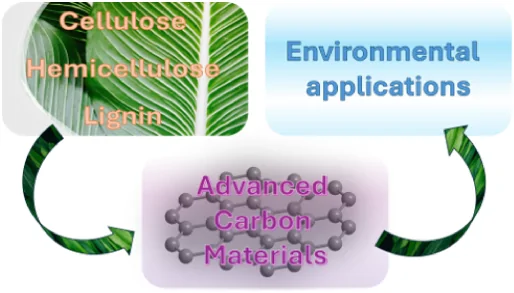

## 1 Introduction

The adoption of renewable energy sources is essential to achieving sustainable development. Biomass has attracted significant attention, because it enables energy production in a renewable and sustainable manner. It consists of nonfossilized organic matter generated through natural or induced biological processes. Biomass offers a wide variety of resources and serves multiple purposes for humanity, as it is a key raw material in various chemical industries, including energy production. Moreover, biomass production systems regenerate quickly, making them an abundant renewable resource that can be utilized to obtain a range of products, materials, and carbon-based fuels.^1^ Within the energy sector, biomass transformation is considered a renewable process since its energy content originates from solar energy captured during photosynthesis and stored in the organic molecules formed.^2^

Throughout history, humans have relied on biomass as an energy source for daily activities. However, with the increasing fossil fuel consumption after the Industrial Revolution, biomass was largely displaced and played a minor role in the global energy system.^1^ Nowadays, the world’s heavy dependence on fossil fuels to meet energy demands has led to significant economic and environmental issues. Extracting fossil fuels has become increasingly complex, and the depletion of these resources has increased their costs substantially. From an environmental perspective, fossil fuels are a major contributor to air pollution due to their high emissions of greenhouse gases, which have led to rising global temperatures and severe natural disasters such as desertification and polar ice melting.^3^ Hence, it is essential to explore energy generation technologies and develop processes that utilize renewable resources to produce high-value chemical compounds. This approach will facilitate the transition toward a sustainable energy system based on inexhaustible energy sources. Biomass stands out as one of the most viable alternatives to conventional energy sources and mitigates the environmental issues related to them. Biomass is the only natural resource on Earth that contains both carbon and hydrogen, enabling the production of energy as well as a variety of high-value chemicals.^4^ Biomass has a variety of chemical compositions. Lignocellulose is the most abundant nonedible biomass, since it is the primary constituent of terrestrial plants.^5^

Lignocellulosic biomass primarily consists of three biopolymers, namely, cellulose ((C_6_H_12_O_6_)_n_), hemicellulose (C_5_H_8_O_4_)_n_, and lignin [C_9_H_10_O_3_(OCH_3_)]_n_, that are interwoven at the microscopic level (see Figure 1). The proportions and arrangement of these components vary depending on the plant species. Table 1 compiles the compositions of typical lignocellulosic biomasses.^6^

**Figure 1:**
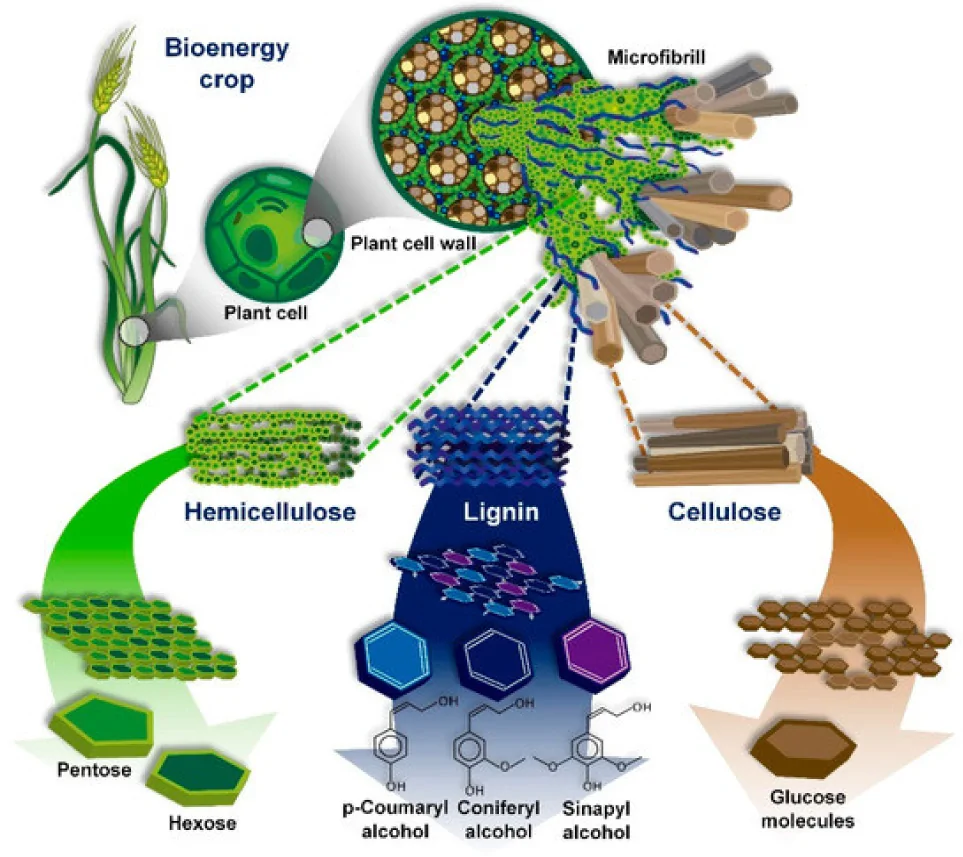
Structure of lignocellulosic biomass and its biopolymers; cellulose, hemicellulose, and lignin. Reprinted with permission under a Creative Commons CC BY 4.0 from^7^. Copyright 2019 MDPI.

**Table 1:** Composition of Typical Lignocellulosic Biomasses^a^

	Components (% in dry basis)				
Biomass	Lignin	Hemicellulose	Cellulose	Ash	^b^Extractives
Hazelnut shell	42.5	29.9	25.9	1.3	–
Hazelnut seed coat	53.0	15.7	29.6	1.4	–
Softwood	28.0	24.4	45.8	1.7	–
Hardwood	21.7	31.3	45.2	2.7	–
Waste material	24.7	29.2	50.6	4.5	–
Tea waste	40.0	19.9	30.2	3.4	–
Sugar cane bagasse	27.2	15.6	49.0	3.6	5.6
Wood (*Eucalyptus globulus L.*)	39.0	7.5	35.6	–	–
Wheat straw	40.4	26.7	28.5	–	–
Rape straw	37.8	37.3	21.2	–	–
Spruce and bark	43.9	24.2	27.0	–	–
Wood (*Eucalyptus globulus*)	21.2	28.5	42.8	0.7	2.7
Sugar cane bagasse	27.79	27.45	39.44	–	–
*Panicum maximum* grass	25.36	26.62	39.87	–	–
*Pennisetum purpureum* grass	22.80	27.03	45.97	–	–
*Brachiaria brizantha* grass	23.09	23.23	43.48	–	–
*Eucalyptus grandis* bark	21.57	18.84	39.54	–	–
Sugar cane bagasse	17.8	–	75.8	2.0	0.9
Sugar cane straw	17.0	–	72.9	4.7	1.4
Coconut fibers	31.84	24.54	38.44	1.41	–
Oil palm empty fruit bunches	35.1	36.6	28.3	–	–
Hemp (*Cannabis sativa L*.*)*	14.68	27.79	50.82	–	–
Pineapple leaf waste	22.0	37.0	30.0	–	11.0
Pineapple stem waste	20.0	34.0	37.0	–	9.0
Pineapple root waste	19.0	32.0	42.0	–	7.0
Wood fiber	16.10	14.86	30.36	–	–
*Pinus radiata*	32.0	21.2	43.8	–	3.2
Corn stalk	15.38	27.93	36.53	–	–
Corn stover	23.2	27.5	32.0	–	–
Poplar wood	24.5	14.0	44.1	–	–
Pine wood	29.5	17.2	41.6	–	–
Wheat straw	25.6	22.2	39.0	–	–
Rice straw	27.5	16.2	34.2	–	–
Moso bamboo	24.5	18.6	41.4	–	–

a Reproduced with permission from Moreira et al.^6^ Copyright 2023 Springer.

b Extractives: terpenes, resins, tannins, carbonates, and silicates.

Lignocellulosic biomass can lead to biofuels, energy, and many chemical products; therefore, it is a sustainable alternative to fossil fuels. Developing efficient processes for the upgrading of this type of biomass is a topic that attracts great interest nowadays.^8–10^

Biomass processing has traditionally focused on generating gaseous and liquid products, with solid byproducts frequently being discarded. Nevertheless, some biomass conversion methods produce solid residues that can be converted to carbon-based materials with a wide range of potential uses. This not only expands the application possibilities but also improves the overall efficiency and value derived from the biomass. In particular, the preparation of carbon materials from lignocellulosic biomass has two main advantages related to pollution management strategy, which are the prevention of potential release and conversion of biomass-based carbon sources into greenhouse gases, and the obtention of nontoxic and environmentally friendly materials with countless applications.^11^ Hence, the valorization of lignocellulosic biomass into advanced carbon materials is inherently driven by the principles of the circular economy. However, evaluating sustainability solely based on the renewable nature of the feedstock introduces a conceptual bias. A comprehensive assessment requires a nuanced analysis of the supply chain, processing energy, and chemical agents used through the lens of life cycle assessment (LCA) and techno-economic analysis (TEA).^12–14^ The upgrading of raw biomass into high-performance carbons often relies on harsh chemical agents, including KOH, ZnCl_2_, and H_3_PO_4_, or energy-intensive modification steps such as heteroatom doping. While these modifications drastically optimize the specific surface area and surface chemistry, their environmental and economic footprints are substantial and should be considered to have a more realistic and overall picture of the sustainability of biomass-derived carbon materials.

The preparation of carbon materials using biomass residues is mainly carried out by gasification and pyrolysis, but hydrothermal carbonization (HTC), which is an environmentally friendly and efficient method for converting biomass into high-value products at moderate temperatures (180–250 °C), has been receiving great attention in the last years.^15–19^

Even though HTC is a well-known thermal treatment, it has not yet been adopted at an industrial scale and is primarily limited to laboratory and pilot-plant scales.^17,20^ The HTC process takes place in a sealed reactor under self-generated pressure, using aqueous media as the reaction environment.^21^ Three main fractions are formed during the HTC process: a solid fraction (carbon-rich material, denoted as hydrochars), a liquid fraction, and a minor gaseous phase.^22^ Raw hydrochars typically exhibit low porosity and poor electrical conductivity. Therefore, post-treatment activation is often required to produce activated carbon materials with enhanced properties that are suitable for various advanced applications.

The selection of the appropriate biomass precursor and the experimental conditions used (temperature, feedstock type, pressure, time, heating rate, water-to-biomass ratio, etc.) will determine the properties of the resulting carbon materials and, hence, their potential applications. Ideally, the precursor should be readily available, economically feasible, and environmentally benign. Additionally, to ensure favorable surface area, structural integrity, and textural properties of the resulting carbon material, a high fixed carbon content and a low ash content are highly desirable. A wide variety of promising precursors have been derived from sources such as wood and woody biomass, herbaceous plants, agricultural residues, industrial biomass waste, and their mixtures. Also, the fabrication of carbon materials can be carried out from different plant components, including cores, stems, shells, peels, flowers, fruits, seeds, stones, husks, and leaves.^23^

The properties of the resulting carbon materials, in terms of pore structure, surface chemistry, and the presence of heteroatoms, can be tailored to design the optimum material for countless fields of research, in which they can act as a catalyst, catalytic support, or adsorbent. However, one of the main disadvantages of using lignocellulosic biomass residues for the preparation of carbon-based materials is the variability and heterogeneity of the raw material—even among different batches of the same biomass feedstock—which sometimes makes it difficult to achieve reproducible results.^24^ The inherent chemical and structural heterogeneity of raw lignocellulosic biomass remains one of the most formidable barriers, preventing its transition from empirical laboratory-scale synthesis to high-end industrial deployment. Biomass feedstocks naturally exhibit profound variations in their cellulose-hemicellulose-lignin ratios, moisture content, and inorganic material content. While these inorganic material fractions can occasionally act as self-activation templates during biochar production or gasification catalysts in further material processing, they are highly detrimental to sensitive frontier technologies such as heterogeneous catalysis and electrochemical applications.^25^ For example, in supercapacitor electrodes or conductive carbon scaffolds, residual alkali and alkaline-earth metals induce severe structural defects, accelerate electrochemical degradation, and increase internal resistance via parasitic side reactions with electrolytes. In catalytic applications, these inorganic impurities frequently block active pore networks, induce sintering of supported active phases, or chemically poison the localized active centers. Consequently, the implementation of rigorous, standardized pretreatment protocols is an absolute prerequisite to ensure batch-to-batch reproducibility and uncompromised performance. Among these strategies, chemical deashing via mineral or organic acid leaching stands out as the primary benchmark for the standardization. Utilizing solutions of HCl, HF (for silica-rich precursors like rice husk), and HNO_3_ allows for the selective protonation and dissolution of the cross-linked inorganic salts embedded within the biopolymer matrix. Mechanistically, acid leaching breaks the coordination and electrostatic bonds anchoring metallic cations to the carboxylic and phenolic oxygen groups of hemicellulose and lignin. This process not only purges the ash fractions but also induces partial hydrolytic deconstruction of the amorphous hemicellulose pathways. This initial deconstruction increases the accessibility of the crystalline cellulose cores to subsequent activation agents.^26^ These standardization protocols transform highly unpredictable agricultural and industrial biowastes into structurally predictable, chemically pure, and high-purity carbon platforms capable of meeting the requirements of advanced applications.

Aside from hydrothermal carbonization, recent literature also includes emerging techniques that rely on nonconventional energy sources to drive chemical transformations, such as microwave-assisted pyrolysis, plasma-enhanced modification, and mechanochemical processes. In the case of microwave-assisted pyrolysis, unlike conventional pyrolysis, which relies on relatively inefficient conductive and convective heat transfer from external sources, microwave-assisted pyrolysis operates via dielectric heating. When biomass is exposed to microwave radiation, polar molecules and free ions within the matrix attempt to align with a rapidly alternating electric field. This alignment induces molecular friction and dipole rotation, resulting in rapid, volumetric, and selective heating.^27–29^ In the case of plasma technology, it is a powerful, low-temperature, and solvent-free approach for the surface engineering of presynthesized biomass-derived carbons. It generally utilizes nonthermal (cold) plasma and takes advantage of highly reactive species, including electrons, ions, free radicals, and excited atoms, while maintaining the bulk gas at near-ambient temperatures. When the carbon-based substrate is subjected to the plasma field, these energetic species bombard the material surface, breaking stable C–C bonds and generating localized reactive sites or structural defects without altering the bulk framework.^30,31^ The mechanochemical approach also represents an interesting pathway for the preparation of carbon materials from lignocellulosic biomass, since it bypasses the need for high-temperature thermal treatments or hazardous chemical solvents by utilizing mechanical energy to initiate chemical reactions and structural transformations, hence holding great potential for large-scale production of advanced carbon-based materials.^32–34^

Due to the importance of carbon materials in environmental remediation, energy storage, and catalytic applications, the present review includes representative examples of carbon materials derived from lignocellulosic biomass residues that are used in these environmental applications. The suitability of carbon materials for these applications stems from characteristics such as their exceptional versatility, derived from a unique combination of high surface area, superior electrical conductivity, and chemical robustness. In the realm of environmental remediation, these materials act as powerful adsorbents capable of removing heavy metals, organic dyes, and pharmaceutical micropollutants from wastewater. Transitioning to energy storage, carbon nanostructures such as graphene and carbon nanotubes are fundamental to the performance of lithium-ion batteries and supercapacitors, where they facilitate rapid electron transport and provide the structural integrity needed for thousands of charge–discharge cycles. Furthermore, in catalytic applications, carbon materials serve as an ideal, inert support that maximizes the dispersion of active metal particles or even function as a metal-free catalyst itself, significantly lowering the energy barriers for crucial industrial reactions.

## 2 Lignocellulosic Biomass-Derived Carbon Materials for Environmental Remediation

A wide range of carbon materials derived from lignocellulosic biomass have been successfully utilized for many years^35^ for the removal of hazardous environmental pollutants, including heavy metals, dyes, and pharmaceuticals, whose effectiveness has been reinforced in recent years.^36^ This effectiveness is largely due to the exceptional surface characteristics of the derived carbons, such as their high specific surface area, large pore volume, presence of heteroatoms, and adjustable micro/mesoporous texture within a hierarchical porous network.^37^ Adsorption using these materials remains one of the most common and effective methods for eliminating both industrial and field-related pollutants from air and water,^38^ and the adsorption mechanism is highly selective and critically depends on the specific physicochemical properties of the target pollutant.^39–42^

As for the heavy metals such as As, Cd, Pb, Cr, and Hg, typically originate from industrial processes and pose a significant threat when released into soil and water systems. Due to their severe health implications, considerable efforts have been made to develop techniques aimed at reducing their concentrations in the environment. Among the various remediation approaches, adsorption using porous carbon-based materials has emerged as one of the most efficient and reliable methods for mitigating these toxic metal pollutants, which has been compiled in numerous studies, in which the adsorption performance has been not only related to the properties of the carbon materials, but also to the adsorption conditions, such as temperature, pH value, contact time, and the amount of carbon material.^37^ For instance, Largitte et al. investigated the performance of activated carbons prepared from three lignocellulosic precursors (namely Guava seeds, Tropical almond shells, and Dindé stones) in the adsorption of Pb­(II) under different experimental conditions, in terms of temperature, initial lead concentration, initial pH, and adsorbent dose.^43^

Wang et al. synthesized a biomass-derived porous carbon material using a straightforward pyrolysis method. The obtained material had a high specific surface area and a well-developed porous structure, making it highly effective for the adsorption of heavy metal ions, specifically Pb­(II) and Cd­(II), from aqueous solutions, achieving adsorption capacities of 155.2 mg g^–1^ and 125.6 mg g^–1^, for Pb­(II) and Cd­(II), respectively, attributed to the porous graphene-like structure of the carbon material, which promoted the easy diffusion and immobilization of metal ions.^44^

Not only “bare” carbon materials have been used for this aim, but also composites. For instance, Cui et al. reported the preparation of a magnetic nanocomposite synthesized *via* one-step pyrolysis of iron-impregnated palm fiber, which demonstrated a high adsorption capacity (16.2 mg g^–1^) for the effective removal of As­(III) from contaminated soils.^45^ The mechanisms underlying arsenic removal involve electrostatic interactions, oxidation of As­(III) to As­(V) by reactive oxygen species, and stabilization *via* iron-based nanoparticles present in the composite.

Aside from the porosity of the carbon materials, the presence of heteroatoms has been demonstrated to play a key role in controlling the adsorption of metal ions. For instance, Li et al. developed an effective adsorbent for the removal of Cr­(VI) from wastewater, which was based on S,N,O heteroatom-co-doped porous carbon spheres prepared from starch.^46^ The mechanism proposed for the removal of Cr­(VI) involved physical adsorption, electrostatic interaction, and chemical reduction of Cr­(VI). The physical adsorption was attributed to the high specific surface area of the carbon spheres (2617 m^2^ g^–1^), the electrostatic interaction was related to the nitrogen functional groups, and the chemical reduction of Cr­(VI) was attributed to the participation of surface functional groups, such as C-SH, -NH-, C–H, and C–OH, acting as electron donors.

The adsorption of dyes is also a very relevant topic because of their impact as environmental hazards and their effects on human health.^47^ Hence, numerous studies have been reported in which different technologies for dye removal are covered.^48^ In particular, some studies are exclusively focused on the removal of dyes by carbon materials prepared from lignocellulosic biomass. For instance, Yadav et al. recently reviewed some recent advances in utilizing lignocellulosic biomass materials as adsorbents for textile-dye removal.^49^ They pointed out the main contributors to the dye effluent, which are the textile industry (54%), dying (21%), paper and pulp (10%), leather and paint (8%), dye manufacturing (7%), and other sectors, such as painting, food, cosmetics, plastics, etc.^49^ Not only carbon materials prepared from lignocellulosic biomass have been used as adsorbents for dye removal, but also raw lignocellulosic biomass wastes such as walnut shells,^50^ pine cones,^51^ pineapple stems,^52^ dragon fruit skins,^53^ etc.

Concerning the adsorption of dyes by carbon materials, several studies covering aspects such as the preparation methods, adsorption capacities, kinetics, isotherm models, and regeneration potentials can be found in the literature.

Among all dyes, Methylene Blue (MB) is probably one of the most investigated because it is one of the highest-consuming materials in the dye industry.^54^ For instance, Benmenine et al. investigated the use of activated carbon prepared from date palm fibers for the removal of MB, and the effects of mass, pH, temperature, and contact time on both removal efficiency and adsorption capacity was examined.^55^ In that case, the removal efficiency ranged from 94 to 99%, and the adsorption capacity was between 96 and 487 mg g^–1^.

Razali et al. used mango seeds and peels for the preparation of activated carbon using a microwave-induced ZnCl_2_ activation, which was used for the removal of MB.^56^ In that study, the effects of adsorbent amount (0.02–0.1 g), pH (4–10), and adsorption time (5–15 min) were explored. It was postulated that the adsorption of MB on this biomass-derived activated carbon could be due to interactions such as hydrogen bonding, π–π interaction, pore-filling, and electrostatic attraction, which are schematized in Figure 2.

**Figure 2:**
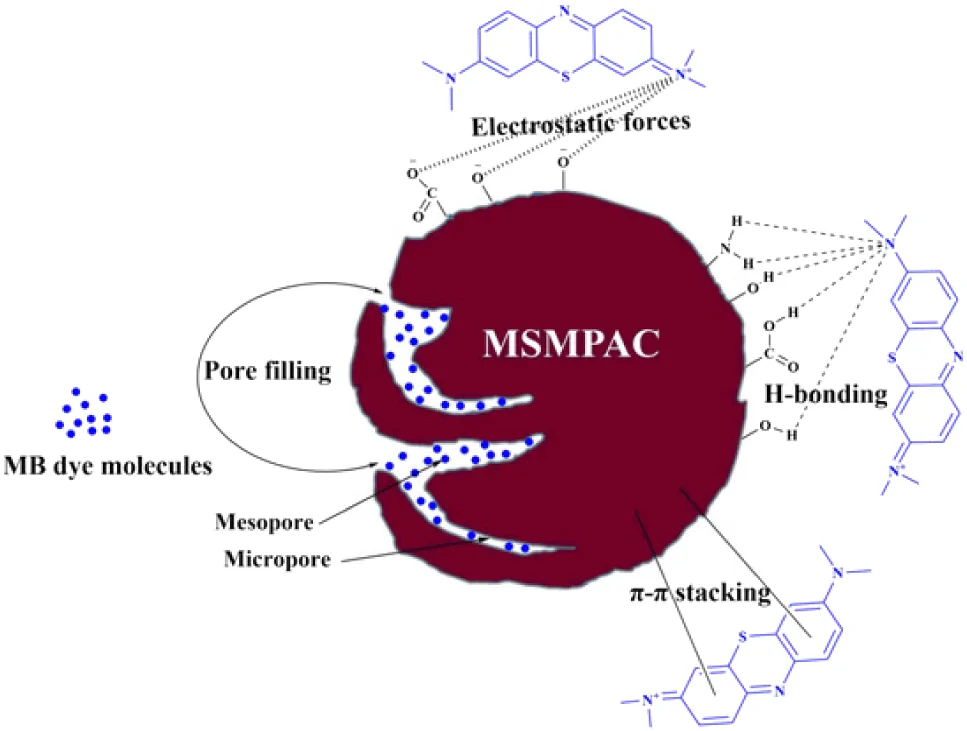
Schematic representation of potential interactions between the activated carbon (denotes as MSMPAC) and MB, including hydrogen bonding, π–π interaction, pore-filling, and electrostatic attraction. Reprinted with permission under a Creative Commons CC BY 4.0 from^56^. Copyright 2022 MDPI.

Wang et al. synthesized activated carbon fibers with a hollow structure and graphite-like crystallites using seed hair fibers of Metaplexis japonicas as the biomass precursor.^57^ A high adsorption capacity (943.4 mg g^–1^) was found in that case, which was attributed to the high specific surface area and rich pores of the hollow fibers, strong electrostatic attraction to the dye due to the presence of surface functional groups, and good contact with dye liquor.

Jia et al. explored the performance of biomass and carbon fibers prepared from biomass, Cortaderia selloana flower spikes, and Cortaderia selloana flower spikes-derived carbon fibers, respectively, for the adsorption of MB.^58^ The adsorption capacity of the raw biomass was almost three times lower than that of the derived carbon material (with a maximum adsorption capacity of 114.3 mg g^–1^). The adsorption performance of both materials was fitted to the Langmuir, Freundlich, Temkin, and Dubinin–Radushkevich (D–R) isotherm models, among which the Langmuir model was indicated to be the most suitable in that case.

MB has also been reported to be efficiently adsorbed in many other lignocellulosic biomass-derived carbon materials, including corn cobs,^59^ loofah sponges,^60^ oil palm fibers,^61^ corn shells,^62^ etc.

The adsorption of other dyes has also been performed by lignocellulosic biomass-derived carbon materials of diverse nature. For instance, the removal of malachite green has been performed using carbon derived from Musa paradisiacal L. leaf (removal efficiency >99%; isotherm model: Redlich-Peterson), Syzyium samarangense (removal efficiency 95.5%; isotherm model: Langmuir), Salacca zalacca (adsorption capacity 69.4 mg g^–1^; isotherm model: Langmuir), Grape grape stalks (removal efficiency 87.7%; isotherm model: Langmuir), Walnut shells (adsorption capacity 154.6 mg g^–1^; isotherm model: Langmuir), Bambusa tulda (removal efficiency 99.73%; isotherm model: Langmuir), etc.^63^ The removal of Rhodamine B was studied with carbon materials obtained from lignocellulosic biomass of diverse nature (Copernicia prunifera, Acrocomia aculeata, and Pinus pinea,^64^ and quinoa straw).^65^ Methyl violet adsorption with carbon materials prepared from canola straw, peanut straw, soybean straw, and rice hull,^66^ and also with durian rind^67^ can also be found in the literature.

Some other studies are not only focused on the adsorption of dyes but also cover the adsorption of heavy metals. For instance, Sherugar et al. demonstrated the applicability of carbon materials prepared from the mangrove fruit of *Rhizophora mucronata* for the simultaneous removal of both heavy metals and dye molecules from wastewater.^68^ Four different dye molecules (i.e., Eriochrome Black T (EBT), Methyl Orange (MO), Malachite Green (MG), and Methylene Blue (MB)), and two heavy metals ions (Pb­(II) and Cd­(II)) were selected in that study. It was indicated that the developed activated carbon had a maximum adsorption capacity of 588.2 mg g^–1^ for EBT and MO, and 666.7 mg g^–1^ for MG and MB. In the case of heavy metals, the activated carbon displayed a removal percentage of 10 ppm of Pb­(II) and Cd­(II) of 99 ± 0.5% and 99 ± 0.4%, respectively, for 50 mg of the activated carbon within 12 min of the time interval.

Lignocellulosic biomass-derived carbon materials have also been fruitfully explored for the removal of pharmaceuticals, such as anti-inflammatory drugs (diclofenac, nimesulide, ketoprofen, ibuprofen, naproxen); antibiotics (norfloxacin, amoxicillin, metronidazole, tetracycline, ciprofloxacin, sulfamethoxazole, sulfadiazine, and sulfapyridine); and other drugs (ranitidine, hydrochloride, carbamazepine, paracetamol). These substances are considered emerging micropollutants because of their nondegradability and continuous release in aquatic environments and sediments.^38^ Some other reviews covering the removal of pharmaceuticals by biomass-derived activated carbons have already been published in the recent literature.^36,69,70^ Hence, only some examples of the adsorption of representative pharmaceuticals by lignocellulosic-biomass-derived carbon materials will be reviewed herein.

Liu et al. prepared a garlic skin-derived porous carbon (denoted as GSP-BC) material with a high specific surface area and oxygen surface functional groups, which showed an excellent adsorption capacity (1911.8 mg g^–1^) for tetracycline hydrochloride (TCH).^71^ It was speculated that, in that case, the adsorption was governed by both physical and chemical interactions (see Figure 3) related to pore adsorption and surface oxygen functional groups, respectively. The recyclability of the adsorbent was assessed by performing 4 consecutive cycles, and the adsorption capacity decreased from 1588.9 mg g^–1^ to 993.3 mg g^–1^. Tetracycline antibiotics were also adsorbed by other lignocellulosic biomass-derived carbon materials, which showed lower adsorption capacities. For instance, Fan et al. prepared activated carbon from hazelnut shells for the adsorption of tetracycline (TC), oxytetracycline (OTC), and chlortetracycline (CTC).^72^ The maximum adsorption capacities found for that material followed the order OTC (321.5 mg g^–1^) > CTC (313.5 mg g^–1^) > TC (302.9 mg g^–1^). Martins et al. used macadamia nut shells for the removal of tetracycline,^73^ and the adsorption capacity was 455.3 mg g^–1^. Jang et al. prepared a biochar from alfalfa,^74^ which had an adsorption capacity of 302.4 mg g^–1^. The same authors studied the adsorption of TC by *Pinus taeda*-derived activated biochar, which had an adsorption capacity of 274.8 mg g^–1^.

**Figure 3:**
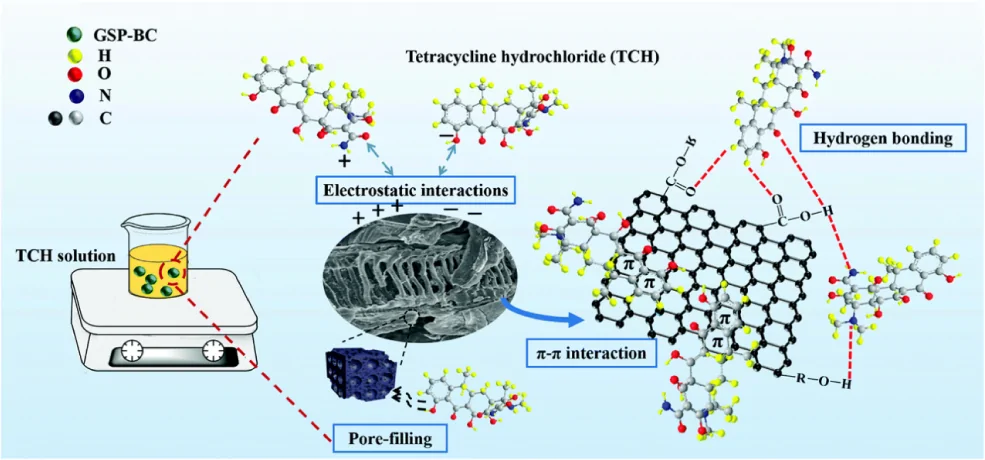
Schematic diagram of the adsorption mechanism of TCH on GSP-BC. Reprinted with permission from^71^. Copyright 2019 Royal Society of Chemistry.

Other antibiotics, such as sulfonamides, have also been removed using carbon materials derived from lignocellulosic biomass wastes. For instance, the competitive adsorption of sulfamethazine, sulfamethoxazole, and sulfathiazole was the objective of Ahmed et al.^75^ They prepared a functionalized biochar from eucalyptus wood, which contained phenolic, carboxylic, ketonic, and isocyanate groups, among others. It was found that the maximum adsorption capacity for sulfathiazole, sulfamethoxazole, and sulfamethazine, respectively, was 45.2 mg g^–1^, 28.3 mg g^–1^, and 20.7 mg g^–1^. Geng et al. studied the behavior of a biochar prepared from walnut shells as an adsorbent for sulfadiazine, sulfamethazine, and sulfachloropyridazine removal, showing and adsorption capacity of 32, 46, and 40 mg g^–1^, respectively.^76^ Huang et al. checked the adsorption capacity of a pristine and ball-milled biochar prepared from bagasse (BG), bamboo (BB), and hickory chips (HC) for sulfamethoxazole and sulfapyridine.^77^ Díaz et al. reported the preparation of activated carbon derived from grape seeds using KOH, FeCl_3_, and H_3_PO_4_, and their application toward the sulfamethoxazole adsorption.^78^ It was found that the material prepared with KOH was more efficient for the adsorption of sulfamethoxazole, showing an adsorption of 650.8 mg g^–1^. Many more lignocellulosic biomass sources have been used in the preparation of carbon materials used in the removal of sulfonamides, which include spent coffee ground,^79^ bamboo biomass,^80^ coconut shell,^81^ etc.

Quinolones are another class of antibiotics that have also been widely investigated in adsorption studies. For instance, Yi et al. reported the development of biochars derived from rice husk and wood chips, which were used in the removal of levofloxacin.^82^ It was found that both the biomass precursor and the temperature used in the synthesis of the biochars influenced the adsorption capacity. Higher temperatures gave rise to materials with higher adsorption capacityies for both rice husk- and wood chip-derived biochars (7.8 mg g^–1^ and 5.0 mg g^–1^, and 4.3 mg g^–1^ and 1.4 mg g^–1^, for the materials prepared at 600 °C and 300 °C, respectively). Corn stalks were used by Wang et al. for the preparation of activated magnetic biochar, which were used for the removal of norfloxacin, reporting an adsorption capacity of 7.6 mg g^–1^.^83^ Norfloxacin removal was also studied by Guan et al. in their work on aged hydrochars prepared from rice husk and corn stalk.^84^ The aging methods employed included chemical aging (H_2_O_2_ oxidation and HNO_3_/H_2_SO_4_ acidification) and physical aging (high temperature and freeze–thaw cycles). The aged hydrochars were used in the adsorption of that model antibiotic in the presence and absence of Cu­(II). Ouyang et al. reported the preparation of H_3_PO_4_-activated biochars derived from peanut shells, corncob, and cotton for the removal of ciprofloxacin.^85^ Other lignocellulosic biomass sources utilized in the preparation of carbon materials for the removal of quinolones include garlic peel,^86^ *Prosopis juliflora*,^87^ apple branches.^88^

## 3 Lignocellulosic Biomass-Derived Carbon Materials for Energy Applications

The development of energy storage and generation devices is essential to harness efficiently renewable energy sources, because some of them are asynchronous energy sources. Therefore, there is a need to store energy due to fluctuations of electricity generation, allowing for coupling the generation with electricity demand, even when these clean resources are in short supply or unavailable. At this point, it is important to be able to develop efficient energy storage systems to successfully tackle this concern. Storage infrastructures based on batteries, fuel cells, and electrochemical capacitors (also named supercapacitors) might collect electrical energy and deliver it when necessary. Carbon materials play a key role in the manufacture of electrodes for such devices, whose electrochemical performance is linked to the electrode materials, among others. The design and optimization of carbon materials must not only take into account the electrochemical performance but also their cost-effectiveness. Biomass-derived carbons can be regarded as inexpensive and sustainable alternatives. A comprehensive collection of carbon materials derived from biomass waste have been synthesized for their use as electrodes in supercapacitors and batteries.^89,90^ The properties of the resulting carbon materials can be tailored by modifying the experimental conditions (temperature and time of carbonization/activation treatments, mass ratio of activating agent/precursor, heteroatom-containing compounds, etc.). Among the properties of carbon materials, apparent surface area, pore volume, and pore size distribution are quite important for the adsorption–desorption of ions involved in the formation of the electric double layer or to attain a good dispersion of the active metal phase in the electrocatalysts used in fuel cells. An in-depth analysis of the electrochemical performance of lignocellulosic-biomass-derived carbon materials reveals that their capacitance and ion transport kinetics are strictly dictated by their interconnected porous network and surface chemistry. The natural cellular structures of lignocellulose enable the synthesis of carbons with a well-balanced distribution of micro-, meso-, and macropores. Specifically, macropores (>50 nm) act as ion-buffering reservoirs that minimize mass transfer resistance, while mesopores (2–50 nm) serve as high-speed pathways that accelerate transport kinetics, facilitating rapid ion diffusion even at high current densities. Concurrently, ultramicropores (<1 nm), whose dimensions closely match the desolvation radius of electrolyte ions, maximize the electrochemically active surface area available for electric double-layer capacitance (EDLC).^91^ However, structural morphology alone does not determine the final capacitance; the presence of heteroatoms in the carbon network (O, N, P, etc.) is also relevant to the performance of carbon materials utilized in energy storage devices. These heteroatoms lead to improvements in physical properties (wettability, electrical conductivity, etc.) or might even modify the electronic properties of adjacent atoms (metal or nonmetal), which act as catalytically active centers of electrochemical reactions.^92–95^ This section describes representative examples of biomass-derived carbon materials used in different energy storage devices.

Electrical double-layer capacitors store energy through ion adsorption that takes place at the electrode–electrolyte interface, showing higher stored energy when such ion adsorption processes are favored. Hence, carbon materials are the unique or main component of their electrodes due to their high apparent surface area, high porosity, and adequate surface chemistry, among others. In this sense, Selvaraj et al. prepared activated carbons with high apparent surface area and high porosity development using wood waste carbon blocks (WCB) as precursor.^96^ One-step chemical activation was performed using an activation ratio of 1:3 (WCB:KOH) and different activation temperatures (700, 800, and 900 °C). The resulting activated carbon nanosheets (ACNs) displayed similar apparent surface areas (from 2786 to 2943 m^2^ g^–1^), being much larger than that of the nonactivated precursor. The capacitance values of ACN- 700, ACN-800 and ACN-900 were 340, 469, and 588 F g^–1^ in 6 M KOH, respectively. Supercapacitors based on ACN-900 exhibited a good stability in KOH. Additionally, this supercapacitor was assessed in a neutral electrolyte, attaining an excellent energy density of 56.73 Wh kg^–1^ at 249 W kg^–1^. The differences in the electrochemical performance might be related to total pore volume (V_T_), since *S*_BET_ are similar. Sometimes, it is difficult to attribute the improvement on the electrochemical performance only to a property of carbon material. Liu et al. reported a similar electrochemical behavior in activated carbons derived from waste coffee grounds (ACGs).^97^ Carbonization followed by chemical activation with KOH were performed using different temperatures of activation. They obtained activated carbons with high apparent surface areas using a ratio of 1:4 (precursor:KOH), although the differences were not significant among ACGs for activation temperatures higher than 800 °C. However, the total pore volumes were different among them. ACG4–900 presented the highest V_T_ (1.82 cm^3^ g^–1^) and that ACG sample displayed the best electrochemical response. Furthermore, ACG4–900 was compared to ACG2–900 (precursor:KOH = 1:2). Their apparent surface areas were 3320 and 3116 m^2^ g^–1^, for ACG2–900 and ACG4–900, respectively, while the V_T_ for ACG2–900 sample was of 1.50 cm^3^ g^–1^ and ACG4–900 1.82 cm^3^ g^–1^, with the last one showing a higher mesopore volume (0.47 vs 0.15 cm^3^ g^–1^). Such enhancement of pore volume is in agreement with the better electrochemical performance of ACG4–900. It is important to mention that pore volume is important to the performance of supercapacitor based on biomass-derived carbon, but not less important is a uniform and/or ordered porosity. In other words, a suitable combination of micropores and mesopores could lead to the improvement of the electrochemical performance. At this point, biomass residues could present a drawback, since biomass-derived carbon materials usually possess poor and nonhomogeneous porosity when these are directly carbonized.^98–101^ However, some routes of synthesis lead to carbon materials with higher porosity, which show relative homogeneity and order.^102–105^ Ruiz-Rosas et al. studied the electrochemical behavior of lignin-based hierarchical porous carbons (HPCs) prepared by carbonization of lignin-zeolite mixture. Zeolite Y and BETA were used as templates to synthesize HPCs. L-Y-900 and L-B-900 displayed similar micropore volumes, while L-B-900 presented a higher mesopore volume than L-Y-900. They observed a remarkable capacitance retention at high current density values for HPCs, which was attributed to the interconnected porous structure.^106^ Then, selected HPCs were assessed in a two-electrode cell. L-B-900-based supercapacitors exhibited better performance in terms of energy density than L-Y-900-based supercapacitors when higher power output was required.^107^ Zhang et al. reported a study that dealt with tuning the ratio of micro/mesopores of biomass-derived carbon sheets. Carbonized pecan shells were activated with different amounts of KOH, resulting in porous carbons with different micro/mesopore ratios. The UCS-2 sample (pecan shell/KOH = 2:1) presented 0.82 and 0.35 cm^3^ g^–1^ of micropore and mesopore volumes, respectively. At a higher precursor/KOH ratio (UCS-3), the mesopore volume increased and the micropore volume decreased, which, in turn, caused a decay in gravimetric capacitance compared to UCS-2. They concluded that a balanced ratio of micro/mesopores agreed with the best supercapacitor performance. The interconnected channels enhanced the mass transport rate, attaining high energy at high power density.^108^ Mechanochemistry has also been used to prepare 3D porous carbons (3DPC). Su et al. mixed pine pollen, NaCl, and KOH using ball milling. The milled powder was carbonized at different temperatures (650, 750, and 850 °C) under an inert atmosphere (see Figure 4).^109^ The one-step method resulted in carbon materials with a graphene-like structure that has a high apparent surface area, an appropriate porosity, and good electrical conductivity due to heteroatom-containing biomass.

**Figure 4:**
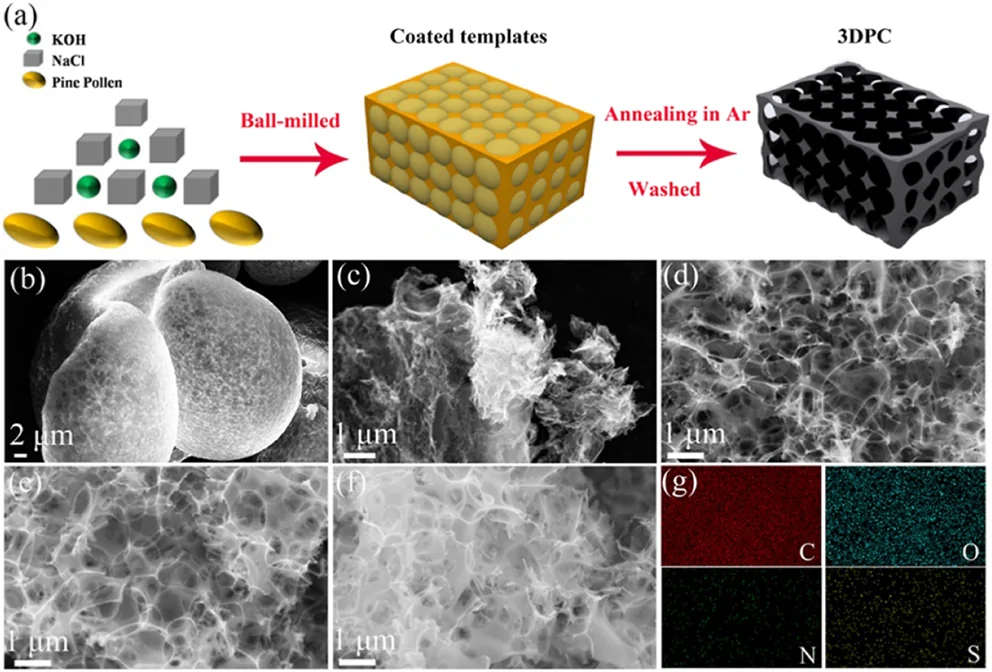
(a) Scheme of 3D hierarchical porous carbon (3DPC) synthesis; SEM images of (b) pine pollen, (c) PC-750 (without KOH), (d) 3DPC-650, (e) 3DPC-750, (f) 3DPC-850, and (g) EDS mapping of 3DPC-750. Reproduced with permission from^109^. Copyright 2022 American Chemical Society.

The sample heat-treated at 750 °C exhibited high specific capacitance and remarkable cycling stability after 10000 cycles (>96%) in 6 M KOH. The supercapacitor based on 3DPC-750 supplied an acceptable energy density at high power density. Furthermore, an all-solid-state symmetric supercapacitor based on 3DPC-750 provided an energy density of 8.41 W h kg^–1^ at a power density of 140.88 W kg^–1^, keeping an energy density of 7.21 W h kg^–1^ at a power density of 3.71 kW kg^–1^. As it was seen, the design of porous frameworks plays a key role in supercapacitor performance, although other properties, such as chemical composition, should be considered. Yuan et al. prepared N-doped interconnected porous carbons (IPC) using an inexpensive lignocellulosic biomass residue (reed, R) and nonlignocellulosic biomass as a nitrogen precursor (chlorella, C).^110^ Through a one-step method in the presence of K_2_CO_3_, nitrogen atoms were introduced into the carbon network, and the nitrogen content of IPC-R_*x*_C_*y*_ ranged from 1.04 to 2.10 wt %. IPC-RC_1.2_ showed a high apparent surface area (1794 m^2^ g^–1^), a pore volume of 1.32 cm^3^ g^–1^, and the highest nitrogen content among those prepared IPCs. IPC-RC_1.2_ exhibited an outstanding electrochemical response. Two-electrode cell based on IPC-RC_1.2_ delivered a high energy density of 23.6 Wh kg^–1^ at a power density of 750 W kg^–1^, and its cycling stability was noticeable after 10000 cycles. The good electrochemical behavior of IPC-RC_1.2_ was attributed to its interconnected porous structure, high *N* content, and high apparent surface area. Yue et al. also reported N-doped porous carbon with uniform porosity (NCSAC) by chemical activation of cornstalk with KOH solution in the presence of urea.^111^ Nitrogen-free samples were prepared in the presence of activating agent (CSAC) and without KOH (CSC) using the same experimental conditions. Unsurprisingly, the electrochemical characterization of as-prepared carbon materials in sodium sulfate demonstrated that the activated samples outperformed CSC in rate capability, owing to their greater porosity development. Regarding activated carbons, they showed comparable rate performance and NCSAC exhibited a higher gravimetric capacitance than the nondoped activated carbon. This improvement was attributed to the incorporation of nitrogen groups. XPS analysis revealed that pyridinic and pyrrolic nitrogen were the principal species. To further explore the potential of NCSAC as supercapacitor electrodes, a NCSAC-based symmetric capacitor was evaluated in the same electrolyte, achieving an energy density of 10.0 Wh kg^–1^ at 249.9 W kg^–1^. Additionally, supercapacitor based on NCSAC showed exceptional capacitance retention (over 99% after 5000 cycles).

Our group has a vast experience in the synthesis of both nondoped and doped carbon materials derived from biomass.^15,92,112–114^ We reported a synthesis method to produce N-doped activated carbon through an organic chemistry route under mild conditions using a hemp residue as precursor.^114^ Our approach consisted of a H_3_PO_4_-assisted hydrothermal carbonization using a low concentration of H_3_PO_4_ followed by heat treatment at 450 °C. The resulting activated carbon (HTC_HR_450) exhibited high porosity development, with an apparent surface area of around 1500 m^2^ g^–1^, and the activation process yield reached nearly 42%, which represents a significant advantage in comparison with conventional activation methods used. Nitrogen functionalization of HTC_HR_450 was carried out using a 2 M NH_4_NO_3_/DMF solution and pyridine, following the procedure reported by Mostazo-López et al.^115^ This N-doped activated carbon was labeled as HTC_HR_450-N. Additionally, HTC_HR_450 was heated at 900 °C, and the carbonized sample was also functionalized. Their nomenclature was HTC_HR_450_TT and HTC_HR_450_TT_N, respectively. Then, the same synthesis protocol was applied using a commercial activated carbon (WV-A1100, MeadWestvaco. Corp.) obtained from wood by conventional H_3_PO_4_ activation. The main nitrogen functionalities were pyridine, amine/amide, and pyrrole groups (N_TOTAL_ = 1.6–1.9 wt %). Such nitrogen species, together with the heat treatment, were involved in enhancing the electrochemical stability and electrical conductivity as well as in minimizing the degradation of carbon material.^116^ Symmetric capacitors based on synthesized carbons were evaluated in organic electrolyte (TEMA-BF_4_/PC). The electrochemical characterization demonstrated that symmetric capacitors based on nitrogen-containing samples displayed good electrochemical performance. The energy density at high power densities for N-doped HTC_HR-based capacitors was comparable to that of capacitors using YP-50F, as shown in the Ragone plot (see Figure 5). Furthermore, the energy efficiency and capacitance retention of these symmetric capacitors were remarkable, closely matching those of YP-50F-based devices. This promising result demonstrates that a straightforward synthesis of nitrogen-containing activated carbon from a renewable precursor can yield competitive symmetric capacitors.

**Figure 5:**
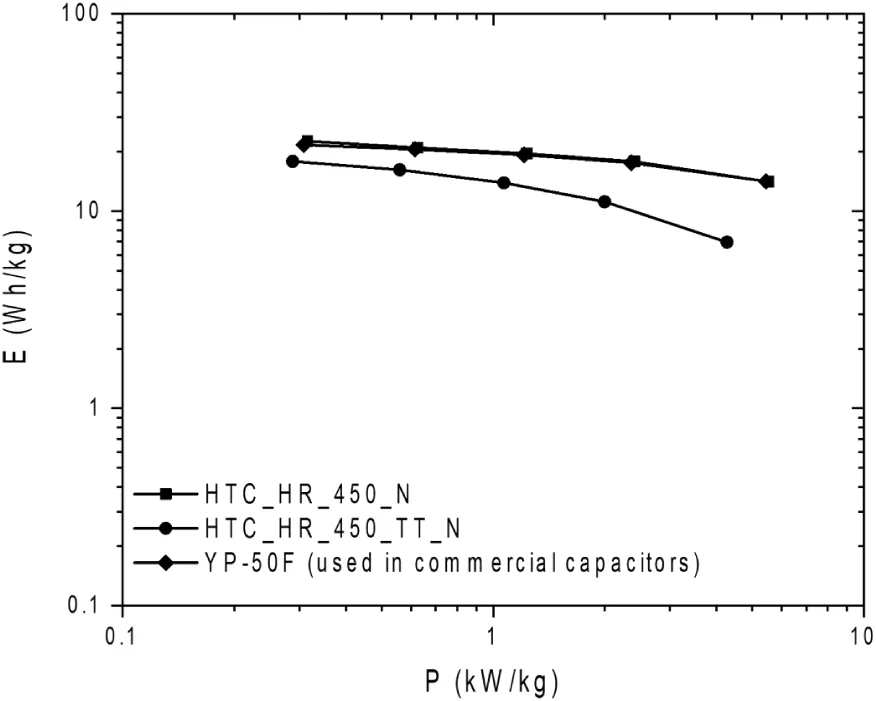
Ragone plot of symmetric capacitors based on heat-treated N-doped activated carbons. Potential window of 2.5 V. Electrolyte: 1 M TEMA-BF_4_/PC. Adapted with permission from^114^. Copyright 2021 Elsevier.

Other heteroatoms have also shown a positive effect on the electrochemical performance of carbon materials.^117^ Wang et al. studied the dual-doping of phosphorus and nitrogen in porous carbon microsheets.^118^ Pomelo peels and diammonium hydrogen phosphate were used as biomass precursor and N,P*-*source, respectively. One-step carbonization was performed using different mass fractions of N,P-source (see Figure 6). Mass fractions of N,P-source ranged from 5 to 40 wt %, and it was observed that apparent surface area increased as mass fraction of N,P-source rose. The specific capacitance exhibited the same trend as *S*_BET_ and the maximum was reached by PPC900-N&P30 sample (30 wt %), which displayed a value of 240 F g^–1^ (at 0.5 A g^–1^) in a three-electrode cell. Symmetric capacitor based on PPC900-N&P30 was assessed in neutral electrolyte (1 M Na_2_SO_4_), supplying an energy density of 11.7 Wh kg^–1^ at a power density of 160 W kg^–1^. The durability test revealed an outstanding cycling stability, showing a capacitance retention of 99% after 10000 cycles at 1A g^–1^.

**Figure 6:**
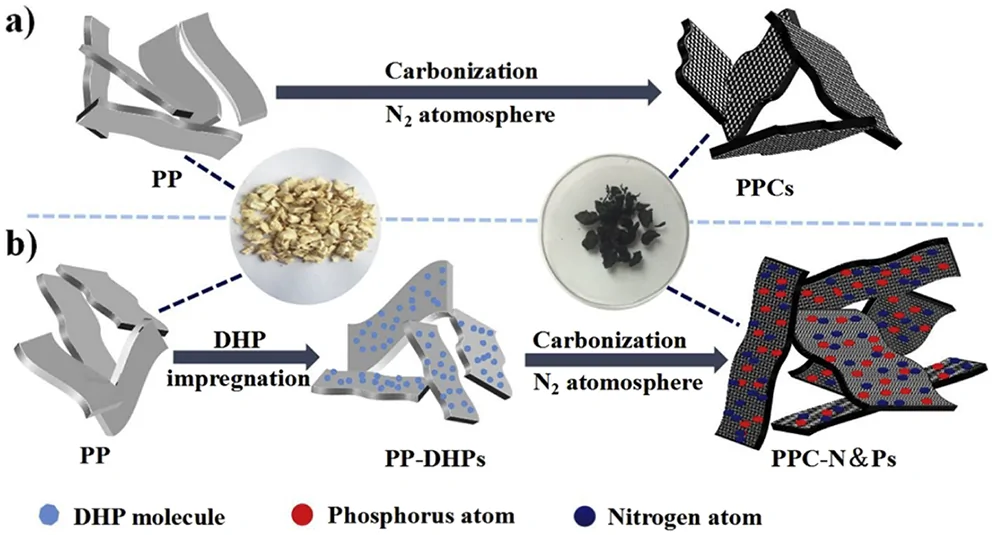
Scheme of synthesis method of N,P-doped porous carbon microsheets and nondoped samples. Reproduced with permission from^118^. Copyright 2018 Elsevier.

Tang et al. reported a synthesis of N,P-doped hierarchical porous carbon derived from bamboo waste through a facile one-step carbonization activation approach using urea and Na_2_HPO_4_ to introduce nitrogen and phosphorus, and K_2_CO_3_ as the activating agent.^119^ Both nitrogen and phosphorus contents were adjusted to assess the effect in the electrochemical performance. The optimized porous carbon (NP_(1:1)_-HPC) displayed an apparent surface area of 1192 m^2^ g^–1^ and a gravimetric capacitance of 386.6 F g^–1^. Symmetric capacitor based on NP_(1:1)_-HPC delivered an energy density of 15.2 Wh kg^–1^ at a power density of 100 W kg^–1^ and a capacitance retention (C/C_0_) of 97.5% after 10000 cycles. Additionally, zinc-ion hybrid capacitor based on high-performing sample was assembled and its displayed a high energy density of 128.9 Wh kg^–1^ at a power density of 179.9 W kg^–1^, obtaining a C/C_0_ around 97% after 10000 cycles. Wang et al. reported a study in which not only nitrogen and phosphorus were incorporated into the carbon network, but sulfur was also incorporated into the carbon matrix.^120^ N,P,S-doped hierarchical porous carbons (NPS-HPCs) were synthesized using a straightforward method, which consisted of a carbonization of rice husk. The carbonized sample was impregnated with NH_4_H_2_PO_4_, thiosalicylic acid, and KHCO_3_ as activating agent. The incorporation of sulfur modified the pore structure compared to that obtained from N,P-HPC. NPS-HPCs presented a higher *S*_BET_ (1976.8 m^2^ g^–1^) than N,P-HPC and its mesopore volume increased over 13% with respect to N,P-HPC. Furthermore, the incorporation of heteroatoms allows for a faster electron transfer, driving the redox process between Zn^2+^ and C=O/phosphorus species. Such properties are involved in a remarkable electrochemical performance of NPS-HPC-based zinc-ion hybrid capacitor, which supplied an outstanding energy density of 167.69 Wh kg^–1^ (at 106.6 W kg^–1^) and maximum power density of 19.64 kW kg^–1^. Furthermore, 23000 cycles were performed, and the capacitance retention remained constant (99.9%).

It is important to note that biomass-derived carbon materials have also been used in other energy-related devices. Some representative examples are described below. One of the most common uses of biomass-derived carbon materials is as an electrocatalyst for the oxygen reduction reaction (ORR). Liu et al. prepared porous carbon nanosheets obtained from the leaves of water lettuce (N-HPCNSs). They proved that N-HPCNSs presented a hierarchical porous structure and a homogeneous distribution of nitrogen functionalities.^121^ As-prepared N-doped carbon electrocatalysts were assessed in different aqueous electrolytes, and these electrocatalysts exhibited high electrocatalytic activity toward the ORR. This electrochemical response indicated that N-HPCNSs are an alternative to Pt-based electrocatalysts commonly used as cathodes in direct methanol fuel cells and Zn-air alkaline fuel cells, among others. Our group has also prepared biomass-derived carbon for s use as an ORR cathode electrocatalyst for fuel cells. Alemany-Molina et al. synthesized, through a simple method, a set of prominent ORR electrocatalysts from biomass waste (almond shell, eucalyptus, and oceanic Posidonia).^113^ The synthesis entailed hydrothermal carbonization of a mix of biomass precursors, FeC_2_O_4_, and dicyandiamide in water. The resulting biochar was carbonized at high temperatures, obtaining Fe-containing N-doped carbon electrocatalysts. It was observed that the iron phase was well-dispersed. The ORR activity of all electrocatalysts was assessed in an O_2_-saturated 0.1 M KOH solution. The best electrocatalyst derived from almond shells corresponded to Fe (5)-N/AS_900, which attained an onset potential of 0.92 V. Concerning the analogous samples derived from eucalyptus (Fe(5)-N/E_900) and oceanic Posidonia (Fe(5)-N/OP_900), onset values were 0.87 and 0.96 V, respectively. Fe(5)-N/OP_900 not only presented an onset potential close to that of Pt/C electrocatalyst (0.97 V), but also the number of electrons transferred was comparable to Pt/C electrocatalyst. Additionally, ORR stability tests were performed over 500 cycles (0 to 1 V) between in a rotating-ring disk electrode (RRDE). Fe(5)-N/OP_900_used displayed kinetic parameters and selectivity similar to those obtained from the fresh catalyst (see Figure 7).

**Figure 7:**
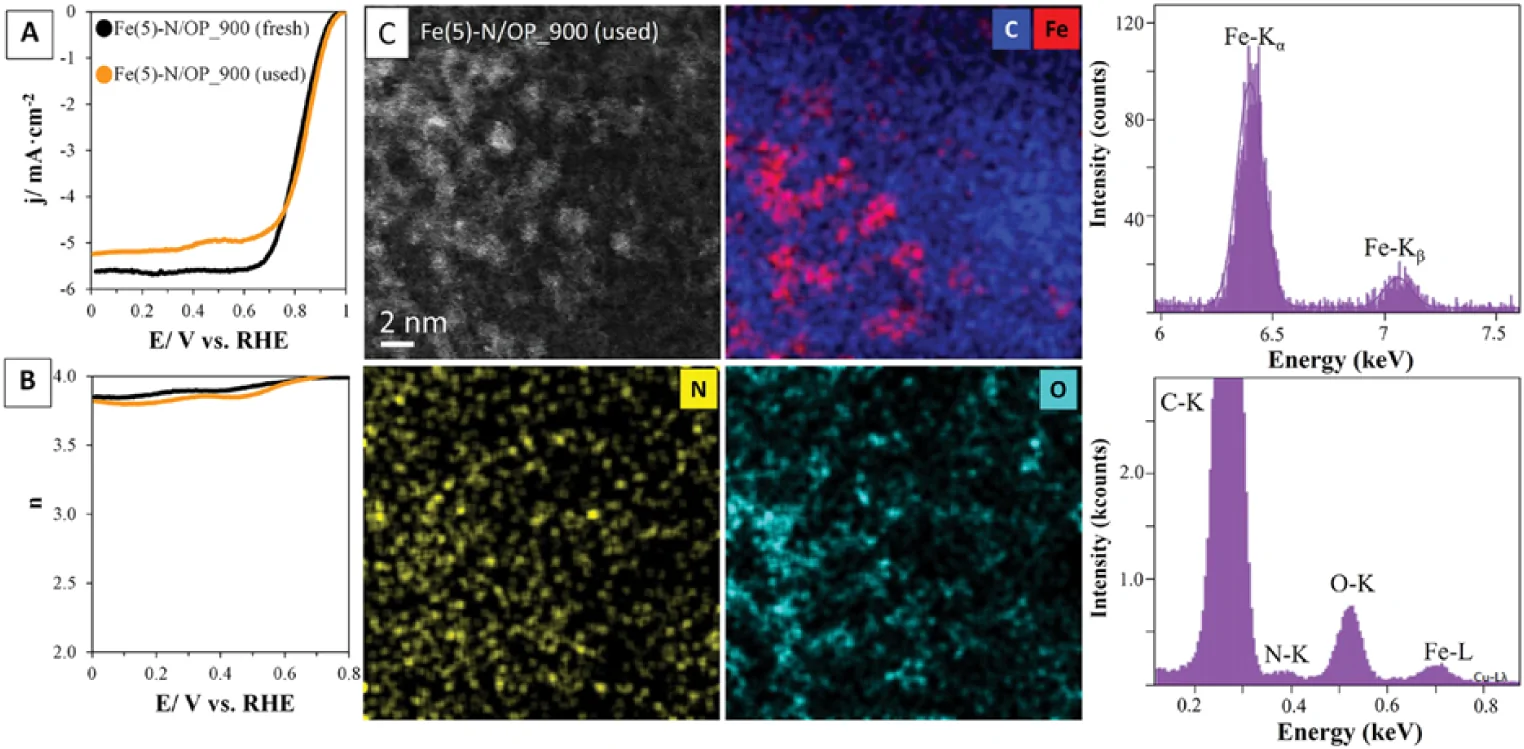
A) LSV curves and B) number of electrons transferred for a fresh and used Fe(5)-N/OP_900 electrocatalyst. (1600 rpm in a O_2_-saturated 0.1 M KOH solution). C) Representative STEM-HAADF-XEDS analysis of the Fe(5)-N/OP_900 used electrocatalyst, along with the corresponding XEDS spectra acquired from the whole area of (c). Reproduced with permission from^113^. Copyright 2025 Wiley.

The high-performing electrocatalyst (Fe(5)-N/OP_900) was evaluated as a positive electrode in a primary zinc-air battery (ZAB). Fe(5)-N/OP_900. From polarization curves, both the current density (150 mA cm^–2^) and maximum power density (59 mW cm^–2^) were comparable to those of Pt/C. The cell voltage was stable for long time and the capacity was 755 mAh g_Zn_^–1^ for Fe(5)-N/OP_900-containing ZABs, while ZABs based on Pt/C showed a capacity of 74 mAh g_Zn_^–1^. Such remarkable electrochemical performance was related to the formation of Fe–N_*x*_ sites. The reproducibility of the proposed synthesis procedure demonstrates its potential as a versatile platform for developing cost-effective electrocatalysts for ORR and other electrochemical reactions. Alemany-Molina et al. also prepared iron-containing N-doped biomass-derived carbon for use as a cathode in alkaline direct methanol fuel cells (ADMFC).^93^ This study was addressed to evaluate the catalytic activity of Fe–N–C sites in two different carbon materials. One of them was obtained from almond shell (AS), and other was prepared by chemical activation of anthracite (KUA). Both electrocatalysts based on AS and KUA (Fe–N/AS and Fe–N/KUA) were comparable in terms of activity and selectivity toward the ORR, using a half-cell configuration. However, their electrochemical performance changed when these catalysts were assessed in a ADMFC device. The Fe–N/AS-containing membrane-electrode assembly exhibited a high-power density close to that of Pt/C (40 wt %) using 1 M MeOH solution. Additionally, the performance of the as-prepared catalysts (Fe–N/AS and Fe–N/KUA) was evaluated at higher concentrations, showing similar performance to those obtained in 1 M MeOH solution, M while the Pt/C-based MEA experienced depolarization at concentrations above 1 M. Concerning the stability, Fe–N/AS electrocatalyst kept high power density after 100 h. The above-mentioned studies pave the way for exploring other electrocatalysts synthesized using different biomass precursors that have demonstrated enhanced structural and electrocatalytic features.

## 4 Lignocellulosic Biomass-Derived Carbon Materials for Catalytic Applications

The unique physicochemical properties of carbon materials make them excellent candidates for use as catalysts and catalytic supports.^122^ The importance of biomass-derived carbon materials in catalysis has been pointed out in a plethora of publications, and the topic has received more attention in recent years. The broad scope of catalysts makes covering all catalytic applications in a single review very complex. Hence, only some selected catalytic applications are covered in this section.

There are numerous studies demonstrating the applicability of lignocellulosic biomass-derived carbon materials in organic reactions, such as reduction and oxidation reactions, coupling reactions, and so on. As for many other applications of carbon materials, the incorporation of heteroatoms is crucial to attain good catalytic performance, being particularly abundant those studies in which nitrogen-containing and phosphorus-containing carbon materials are investigated.

Liu et al. prepared radish-derived N-doped porous carbon, which was used in both the catalytic reduction of 4-nitrophenol and the oxidation of styrene.^123^ Very interesting catalytic results were reported in that study, achieving performances that were comparable to some of those attained by metal–metal-containing catalysts.

Supriya et al. prepared catalysts based on Pd and areca nut kernel-derived carbon nanospheres for the reduction of nitroarenes and the Suzuki–Miyaura coupling of bromoarenes with aryl boronic acids.^124^

Yin et al. prepared P-doped carbon catalysts that had inherent N functionalities, using waste peanut shells as the starting material, and they were used as catalysts for the selective aerobic oxidation of alcohols.^125^ These materials displayed better performance than those of the single-element-doped carbon for the oxidation of benzyl alcohol, and the materials displayed good recyclability during, at least, four reaction cycles.

Rodríguez-Mirasol and Cordero’s group has vast experience in the preparation of P-containing biomass-derived carbon materials in catalytic reactions. One of the catalytic reactions in which such P-doped carbons were used was the dehydration of alcohols. It was reflected, for example, in the study of Valero-Romero et al., who explored the suitability of P-containing catalysts derived from olive stones for the dehydration of methanol to dimethyl ether, a promising diesel substitute. It was found that P groups endowed the carbon materials with high oxidation resistance, surface acid, and redox sites, and the resulting catalysts had a steady-state methanol conversion of 20% at 300 °C and 95% of selectivity toward dimethyl ether.^126^ Torres-Liñan studied the performance of a biomass-derived P-containing carbon doped with zirconium for the production of dimethyl ether from methanol dehydration.^127^ Bedia et al. used P-containing hemp-derived carbon material for the dehydration of 2-propanol to propylene.^128^ The same group also used P-containing lignocellulosic biomass-derived carbon materials for the preparation of other products of interest for biorefinery processes, such as solketal and γ-valerolactone.^129^

Concerning coupling reactions, Patel et al. prepared catalysts based on Pd nanoparticles and activated mesoporous carbon derived from rice husk, which were used for the Mizoroki-Heck, Suzuki, and Sonogashira cross-coupling reactions.^130^ The carbon materials obtained had hollow mesoporous structure with an average pore size of 3.5 nm and a surface area of around 1020 m^2^ g^–1^. It was observed that the Pd catalysts prepared in that study were active in the three studied reactions, and they were also reusable for at least ten times. Pine needles were used by Ferlin et al. to prepare Pd-based catalysts for the Sonogashira reaction.^131^ The reaction was operated in continuous flow conditions, and the catalysts were assessed for 15 days, during which they showed constant efficiency and no significant metal leaching.

Kempasiddaiah et al. reported the synthesis of mesoporous carbon from radish leaves, which were used for the preparation of Pd catalysts for the Suzuki–Miyaura cross-coupling reaction.^132^ Those carbons were first oxidized with H_2_O_2_ to increase the density of surface hydroxyl groups, and then they were amine-functionalized by treating the surface of the material with *N*-[3-(trimethoxysilyl)­propyl]­ethylenediamine (TPEA). After that, Pd was incorporated, and the resulting catalyst was denoted as BC-TPEA@Pd (see Figure 8). The obtained catalyst exhibited activities toward the Suzuki–Miyaura cross-coupling reaction comparable to those found in the literature for other materials, and it was reusable during six cycles.

**Figure 8:**
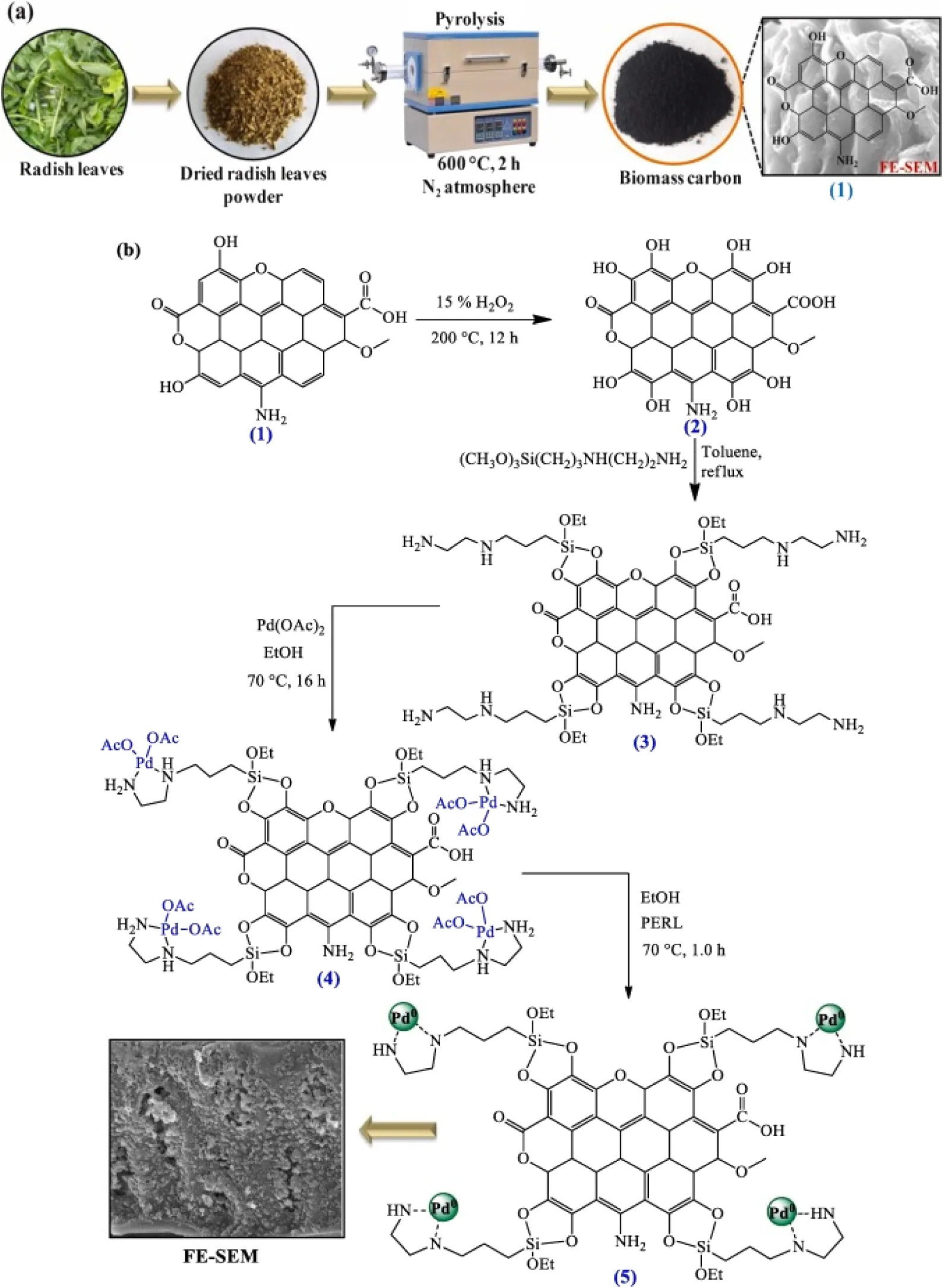
Plausible reaction scheme for the conversion of (a) radish leaves to biomass-derived carbon (1) and (b) synthesis of BC-TPEA@Pd catalyst (5). Reproduced with permission from^132^. Copyright 2021 Elsevier.

Lignocellulosic biomass residues have found application for the preparation of carbon materials that have been used in other interesting catalytic applications. For instance, Godino-Ojer et al. reported the use of Hedychium gardnerianum for the synthesis of activated carbon, with a BET area of 2197 m^2^ g^–1^ and acidic surface functional groups, via pyrolysis and surface phosphonation. Those materials were able to catalyze the synthesis of quinoxalines from 1,2-diamines and α-hydroxyi ketones, under aerobic conditions.^133^

Other authors emphasized the valorization of biomass residues by using biomass-derived carbon-based materials as catalysts for the production of biomass-derived molecules.^134^ For instance, Li et al. prepared activated carbon from coconut shell and investigated its performance for the production of valuable chemicals, such as furans and phenols.^135^ The developed materials were analyzed to relate the physicochemical properties of the catalyst with the distribution of the different products. The fast pyrolysis of corncob at 350 °C selectively produced furans and phenols, which was related to the presence of tailored acid sites on the surface of the catalyst, which was due to the presence of graphitic carbon with phenolic, alcohol, and ether components.

Chen et al. prepared N-doped biochar catalysts with different nitrogen contents using bamboo and several NH_3_ concentrations (10 vol%, 30 vol%, and 50 vol%), which were used for the generation of phenols, such as 4-vinylphenol and 4-ethylphenol, from the catalytic pyrolysis of bamboo.^136^ It was observed that the content of phenols in the bio-oil attained from the catalytic pyrolysis of bamboo significantly increased upon the utilization of those N-doped biochars (Figure 9). In the absence of biochar, the phenol content was 47%, and it increased to 59% when N-free biochar was used. It was seen that the presence of N-containing biochar increased the relative content of phenols in the bio-oil, reaching a maximum value of 82% for biochar-N30. It was suggested in that study that the effect of N-doped biochar not only depended on active N-containing groups and the apparent surface area of the materials but also on the distribution of active species on the surface of the biochars.

**Figure 9:**
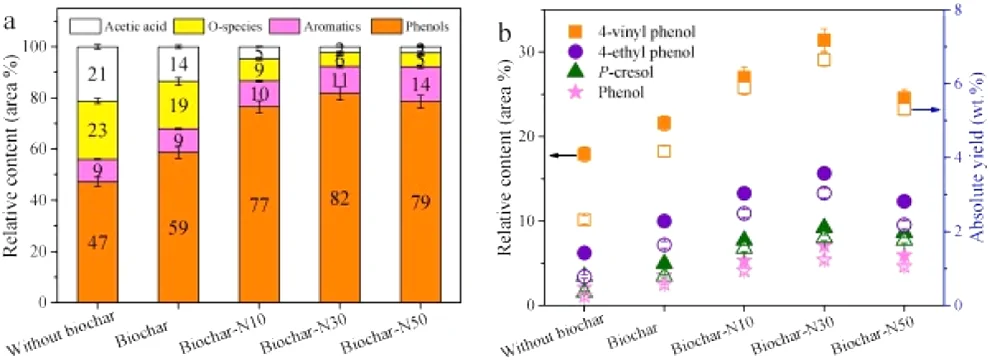
Bio-oil compositions of catalytic pyrolysis (a) and major phenols distribution (b). Solid represented relative content; open represented absolute yield. Biochar-N10, Biochar-N30, and Biochar-N50 stand for the catalysts prepared with the different concentrations of NH_3_ (10 vol%, 30 vol%, and 50 vol%). Reproduced with permission^136^. Copyright 2020 Elsevier.

The use of lignocellulosic biomass-derived carbon materials in catalytic applications, addressed to the conversion of biomass sources into valuable chemicals also has relevance in an ideal sustainable scenario. Kobayashi et al. reported, for the first time, the valorization of lignocellulosic biomass using a biomass-derived heterogeneous catalyst.^5^ In their study, eucalyptus was used to produce a carbon-based catalyst for the depolymerization of lignocellulose. Abdu et al. developed a facile one-pot synthesis of carboxylate carbon-based catalyst from eucalyptus, which was used in the hydrolysis of eucalyptus to xylose and glucose, attaining yields of 95.1% and 81%, respectively, at the experimental conditions used in that study.^137^ Tinh et al., who used corncob, extracted the cellulose fraction and prepared cellulose-derived magnetic iron oxide/sulfonated graphene oxide-like material to be used in the conversion of hemicellulose to furfural.^138^ The materials developed in that study showed a furfural yield of 55.05%, xylose conversion of 97.80%, and furfural selectivity of 57.2% with the optimum experimental conditions. In addition, they were stable for six cycles, and were recovered because of the presence of Fe_3_O_4_ particles grafted onto the surface.

Our research group has extensive experience in developing lignocellulosic biomass-derived activated carbon prepared from different residues, which have also been used for the conversion of biomass-derived molecules such as formic acid. We have recently reported that those materials have great potential for important environmental applications, such as the dehydrogenation of formic acid addressed toward the production of hydrogen. In a first study, we prepared activated carbon from a hemp residue using a H_3_PO_4_-assisted hydrothermal carbonization followed by an activation step. The resulting activated carbon was subsequently functionalized with nitrogen groups, and Pd-based catalysts were prepared using both N-free and N-containing supports.^139^ The activity of those materials toward the dehydrogenation of formic acid in the liquid phase was evaluated, showing great performance and excellent catalytic activity along the reaction cycles. The best performance sample had an initial TOF 8365 h^–1^, expressed per surface Pd atom, but its most remarkable aspect was its excellent stability, since its initial activity was preserved during 12 consecutive reaction cycles. The interesting results attained in that study motivated us to further explore the potential of hemp residues for the preparation of activated carbons as supports for Pd-based catalysts used in the dehydrogenation of formic acid. In a subsequent study, we investigated the effect of the properties of the supports by preparing a set of catalysts in which the surface chemistry of the supports was modified by carried carrying out a thermal treatment at high temperatures (i.e., 900 °C).^140^ Materials with nitrogen functionalization and the combination of both thermal treatment and nitrogen functionalization were also prepared. It was observed that the removal of surface oxygen groups resulting from the thermal treatment of the carbon materials, and the introduction of nitrogen groups, favored the interaction between the surface of the catalysts and the formic acid molecules, which gave rise to active and stable catalysts with leaching and sintering resistance.

In subsequent studies, we developed activated carbon from hard lignocellulosic biomass (i.e., almond shell), which was used as support for PdAg bimetallic nanoparticles.^141,142^ We first checked how the experimental protocol used in the synthesis affected the performance of the PdAg bimetallic samples.^141^ It was observed that, unlike monometallic Pd catalysts, which could be *in situ* prepared under reaction conditions with the H_2_ generated from the dehydrogenation of formic acid, PdAg catalysts needed a reduction step prior to the catalytic reaction. The positive role of the incorporation of Ag into the nanoparticles was demonstrated in that study, while the optimum composition of PdAg samples was determined in a second work.^142^ The performance of the sample was evaluated by monitoring the gas evolution profiles attained from the decomposition of formic acid (HCOOH ↔ H_2_ + CO_2_), and the effect of the composition of the PdAg catalysts in N-free (denoted as AS catalysts) and N-containing (denoted as N-AS catalysts) catalysts was checked. Figure 10 displays the gas evolution profiles achieved by those samples when the reaction was carried out at 75 °C.

**Figure 10:**
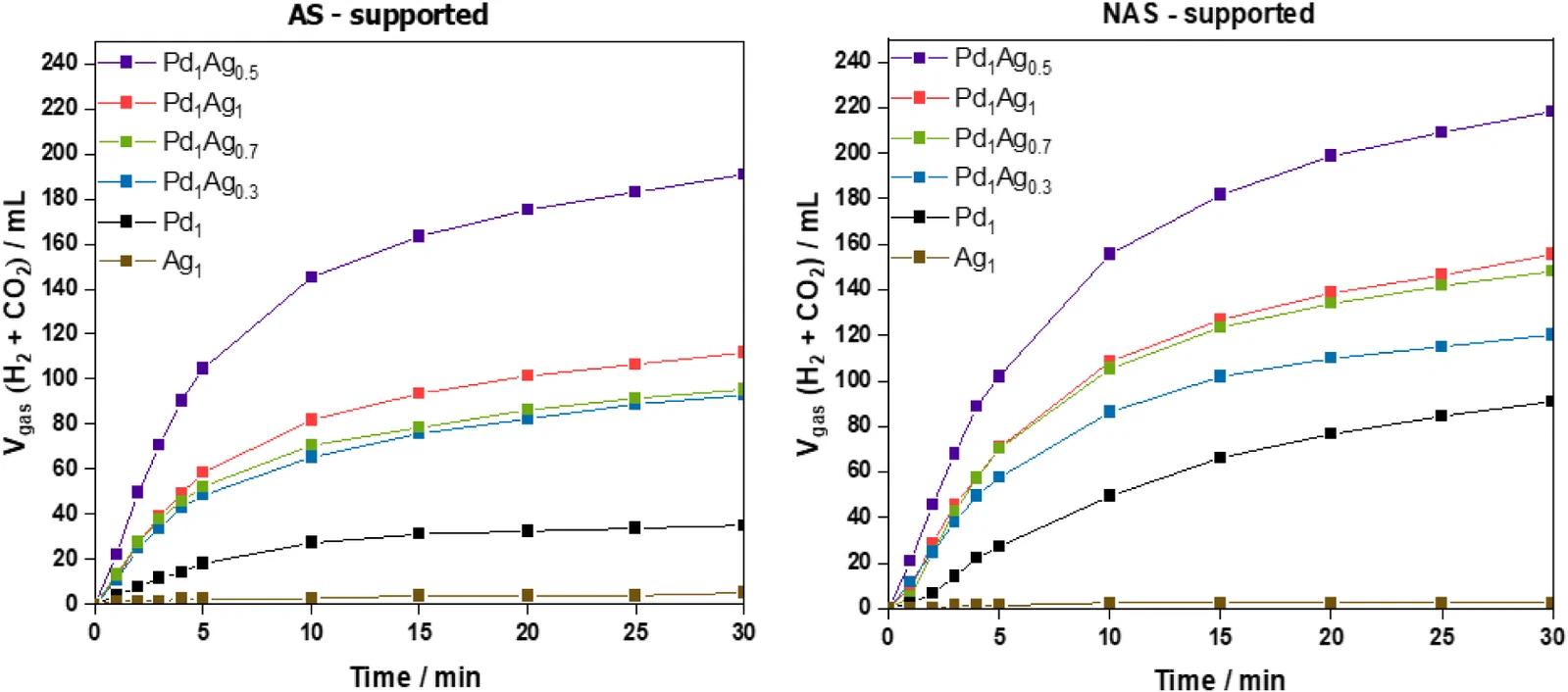
Gas evolution profiles achieved by AS-supported and N-AS-supported catalysts at 75 °C. Reprinted with permission under a Creative Commons CC BY 4.0 from^142^. Copyright 2024 Wiley.

The results attained demonstrated the positive role of the incorporation of nitrogen functional groups in the samples (N-AS set of samples) and also the enhancement achieved upon the incorporation of Ag in the nanoparticles. It was seen that, under the experimental conditions used in that study, the samples with a Pd/Ag molar ratio of 1/0.5 were the best-performing among those investigated for both N-free and N-containing materials (samples denoted as Pd_1_Ag_0.5_/AS and Pd_1_Ag_0.5_/N-AS, respectively). The good activity demonstrated by that sample was related to the electronic properties of Pd species and its impact on the different reaction steps.

Duan et al. also prepared catalysts for the dehydrogenation of formic acid.^143^ In that case, the materials were based on Pd–Co_2_P nanoparticles loaded on N-doped carbon microsheets derived from poplar catkins. The most remarkable aspect of the performance of those materials was their stability, since they preserved 97.6% of the initial activity in terms of hydrogen production during seven consecutive cycles. Shi et al. also prepared poplar catkin-derived Pd-based catalysts for the dehydrogenation of formic acid, materials that also attained good stability during three consecutive reaction cycles.^144^ Peanut shells and melon seeds were used by Cao et al. as starting materials for the synthesis of microporous carbon, which was used as a support of Pd catalysts used in the dehydrogenation of formic acid.^145^

## 5 Summary Remarks

The conversion of lignocellulosic biomass to advanced carbon materials represents a pivotal paradigm shift in sustainable environmental technology and materials science. Composed of an intricate natural matrix of cellulose, hemicellulose, and lignin, this abundant, renewable, and cost-effective biopolymer precursor offers distinct structural advantages over its traditional synthetic counterparts. Its valorization adheres to the principles of a circular economy by upcycling agricultural and industrial waste, thereby mitigating the anthropogenic environmental impacts. At a macromolecular level, the intrinsic structural hierarchy and diverse surface oxygen functionalities of lignocellulose provide a highly adaptable platform for targeted modifications. The performance of these resulting biomass-derived carbon materials across environmental remediation, energy storage, and catalysis is directly linked to microstructural features, such as pore size distribution, graphitization degree, and surface chemistry, which, in turn, depend on the biochemical composition of the selected lignocellulosic precursor and the experimental conditions used in the synthesis. Hence, holistic control of these parameters is what enables the tailored design of biomass-derived carbon materials for specific fields. The proper selection of these parameters will afford carbon materials with a well-balanced distribution of micro-, meso-, and macropores that govern mass transfer kinetics and active site accessibility. While interconnected macropores and mesopores mitigate diffusion limitations by serving as high-speed transport pathways and ion-buffering reservoirs, optimized micropores provide the high specific surface area necessary for the physical trapping of molecular pollutants, the accumulation of electrostatic double-layer charge, or the high dispersion of catalytic phases. Simultaneously, the material surface chemistry, which is initially defined by oxygen-containing functional groups and further tailored via the strategic doping of heteroatoms, such as nitrogen, sulfur, or phosphorus, modulates the localized electron density of the carbon lattice. This chemical tuning dramatically enhances surface polarity and interfacial wettability while concurrently introducing highly reactive sites capable of driving coordinate covalent complexation, reversible faradaic redox reactions for pseudocapacitance, and targeted electron-transfer processes in heterogeneous catalysis.

However, the transition from empirical laboratory-scale success to industrial deployment is currently bottlenecked by critical challenges, most notably feedstock heterogeneity, scalability of precise functionalization protocols, and batch-to-batch reproducibility. To circumvent these limitations, future research trajectories must prioritize the optimization of synthesis pathways, the engineering of advanced hybrid materials, and the execution of rigorous life-cycle assessments (LCA) and technoeconomic analyses (TEA). Consequently, concerted interdisciplinary efforts are imperative to bridging the gap between fundamental laboratory innovations and scalable, technologically viable solutions for global environmental sustainability.
